# Stroke Related Knowledge, Prevention Practices and Associated Factors Among Hypertensive Patients at University of Gondar Comprehensive Specialized Hospital, Northwest Ethiopia, 2021

**DOI:** 10.3389/fneur.2022.839879

**Published:** 2022-04-19

**Authors:** Fkrte Kebede Woldetsadik, Tesfa Kassa, Workagegnehu Hailu Bilchut, Alemu kassaw Kibret, Yisak Girma Guadie, Getachew Azeze Eriku

**Affiliations:** ^1^Department of Physiotherapy, School of Medicine, College of Medicine and Health Science, University of Gondar, Gondar, Ethiopia; ^2^Department of Internal Medicine, School of Medicine, College of Medicine and Health Science, University of Gondar, Gondar, Ethiopia

**Keywords:** stroke, knowledge, practice, hypertension, Ethiopia

## Abstract

**Introduction:**

Stroke is a global health problem, causing significant morbidities and mortalities in both developing and developed countries. Patients living with chronic diseases like hypertension are at a high risk of stroke. Approximately 80% of strokes could be prevented with necessary preventive practices. There is scarce evidence in the preventive practices in Ethiopia, particularly in the study area. Therefore, this study aimed to assess knowledge and prevention practices related to stroke among hypertensive patients at the University of Gondar comprehensive specialized hospital, northwest Ethiopia.

**Methods:**

An institution-based cross-sectional study was conducted among 393 hypertensive patients at the University of Gondar Comprehensive Specialized Hospital from May 1st to June 30th, 2021. The study participants were selected by a systematic random sampling technique. A semi-structured and interviewer-administered questionnaire was used to collect data. The data was entered into EPI Info version 7.2.1 and analyzed with SPSS version 23.0. Binary logistic regression analyses were undertaken to identify associated factors. The level of significance was determined using the adjusted odds ratio (AOR) with its 95% CI at a *p*-value of 0.05.

**Results:**

Participants in this study had adequate knowledge of stroke and good prevention practices in 40.7% (95% CI: 35.9, 45.5) and 51.7% (95 CI: 46.8, 56.5) of cases, respectively. Attending secondary education and above 4.6 (95% CI: 2.08, 10.17), knowing someone who has had a stroke 13.17 (95% CI: 7.3, 23.77), and physical activity 4.05 (95% CI: 2.23, 7.36) were all significantly associated with adequate stroke knowledge. Furthermore, educational status (attending primary education 2.61 (95% CI: 1.44, 4.73) and secondary education and above 3.75 (95% CI: 1.99, 7.05), being an urban dweller 9.65 (95% CI: 5.04, 18.44), duration of hypertension 1.9 (95% CI: 1.15, 3.14), knowing someone with a stroke 2.27 (95% CI: 1.30, 3.93), and physical activity 1.76 (95% CI: 1.03, 3.01) were associated with good stroke prevention practices.

**Conclusion:**

The proportion of participants with good-related knowledge and prevention practice is relatively good.

## Background

Stroke is the leading cause of significant mortality and disability worldwide (1). Globally, stroke is the second leading cause of death with 6.5 million deaths and the third leading cause of death and disability combined, after neonatal disorders and ischemic heart disease with 143 million disability adjusted life years (DALYs) (2, 3). In 2019, there were 101 million prevalent cases of stroke and 12.2 million incident stroke cases globally (1).

Despite the tremendous progress and improvement in evidence-based stroke management, the burden of stroke has rapidly increased over the past three decades (4) and it has shifted from developed to low- and middle-income countries (4, 5). Several factors have been identified as contributing to this burden shift, including aging population, population growth, and increased burden of cardiovascular diseases, changing disease patterns, rapid socio-demographic and lifestyle changes, and health care (6, 7). Currently, 75.2% of all stroke deaths and 81.1% of the total DALYs lost due to stroke occur in developing countries (5, 8, 9). The mortality rate from stroke in sub-Saharan Africa (SSA) is five times higher compared to developed countries (1, 10). The prevalence of ischemic and hemorrhagic strokes in Ethiopia was 31.5 and 18.3%, respectively, with 50.2% having an undetermined type of stroke (11). Stroke, therefore, has emerged as a major public health priority in developing countries (7).

Stroke has a significant impact on physical, mental, and social wellbeing of the individual life (12). It not only affects the individual, but it also places a caregiver burden on family members, and the direct cost of stroke management and indirect loss of productivity from both the affected individual and their caregivers is enormous, resulting in a high social burden (13, 14).

Although healthcare providers may know the prevention of stroke and control the major risk factors, patients' lack of knowledge about risk factors for experiencing new events of stroke is a contributing factor to poor adherence to medical advice and treatment (15). Whereas, awareness of stroke risk factors and warning signs is important for stroke prevention and seeking care for hypertensive patients, ultimately reducing the occurrence of stroke (16). Patients living with chronic non-communicable diseases like hypertension are at high risk of developing strokes (17). Almost half of the stroke survivors were diagnosed with hypertension, and one fifth of them died in the hospital (18). In Ethiopia, hypertension is the most commonly reported risk factor for stroke, accounting for 37.1% of all stroke risk factors. Hemorrhagic stroke (45.1%) is more prevalent than ischemic stroke (37.6%) (11).

The knowledge of stroke warning signs and risk factors has been found to be important for early treatment of stroke (19) and prevention of stroke, which reduces mortality due to stroke and improves prognosis for stroke survivors (20). To a large extent, stroke is a preventable condition, there are many modifiable risk factors of stroke (21). Prevention strategies that focus on modifiable factors such as hypertension, diabetes mellitus, smoking, unhealthy diet, and sedentary physical activity practices can reduce the stroke burden by up to 80% (22, 23).

Even though early identification of stroke warning signs and risk factors is essential for stroke prevention and early management, there is low level of knowledge in the general population (24, 25). The studies done in Ethiopia investigated only the warning signs, risk factors, and prevention knowledge of stroke (26–28). Furthermore, no research has investigated the stroke prevention practices of hypertensive patients. To fill this knowledge and prevention practice gap, this study aimed to determine the knowledge, prevention practice, and associated factors of stroke among hypertension patients in the University of Gondar comprehensive specialized hospital.

## Methods and Materials

### Study Design, Setting and Period

An institutional-based cross-sectional study was conducted from May 1st to Jun 30th, 2021 at the University of Gondar Comprehensive Specialized Hospital (UoGCSH), Gondar city, northwest Ethiopia. Gondar city is the administrative city of central Gondar zone which is found in Amhara region and located 738 km from Addis Abeba and 175 km from Bahir dar which is the capital city of the region. UOGCSH is a specialized hospital in the Gondar area providing a tertiary level health care services as a major referral center for more than 7 million people from the surrounding areas. The UOGCSH has an outpatient department that provides follow-up services for chronic disease including hypertension, stroke and diabetes.

### Study Population and Eligibility Criteria

Hypertensive patients who have been attending a chronic care follow-up clinic at UOGCSH during the data collection period were included in this study. Patients with hypertension who had previously experienced a transient ischemic attack or stroke, critically ill patients, underlying severe mental illness, or were unable to provide the necessary information on their own were excluded.

### Sample Size Determination and Sampling Procedure

The sample size was determined using the single population proportion formula with the assumption of a 95% level of confidence and a 10% marginal error (d). With the assumption of a 50% proportion of hypertensive patients who have good knowledge and prevention practices toward stroke, there is no similar study on stroke prevention practice done in a similar area.


$$n=\frac{{\left(Za_{/2}\right)}^{2}*P\left(1-P\right)}{d^{2}}n=\frac{{\left(1.96\right)}^{2}*0.5\left(1-0.5\right)}{{\left(0.05\right)}^{2}}=385$$

Where n = the required sample size for the study.

d ^2^ = margin of tolerable sampling error commonly used 0.05.

Z = is 1.96 for 95%.

Among the two-sample sizes calculated, the larger sample size, which is 385, has been selected.

The required sample size is 424, with a 10% non-response rate.

The hospital records showed there were about 250 to 270 hypertensive patients in the outpatient's clinic every week. Thereafter, the skipping interval (Kth) was calculated by dividing the 1-month report by the calculated sample size (1,040/424), resulting in 2. The study participants were selected randomly using a lottery method. Finally, a systematic random sampling technique was used to select all eligible study participants.

### Data Collection Procedure and Tool

The data was collected using an interviewer-administered structured questionnaire adapted from previous literature (14, 26, 27, 29), which contained socio-demographic, clinical, and lifestyle characteristics, stroke knowledge items, and stroke prevention practice items.

The knowledge section comprises 16 questions assessing the warning signs and risk factors related knowledge toward stroke with three possible responses: **“Yes”** or **“No;” “I don't know;”** and scores of 1 or 0 respectively. Participants who answered correctly to 16 stroke knowledge related questions, scored equal to or more than the mean value, were defined as having adequate stroke knowledge (27). The stroke prevention part consists of 9 questions with three possible responses: “**Yes”** or “**No;” “I don't know;”** and scores 1 or 0 respectively. Participants who answered 9 questions correctly or had a score greater than or equal to the mean score were considered to have good stroke prevention practice (27).

#### Usual Alcohol Consumption

A person who drinks alcohol on a regular basis (more than 6 days per week). According to World Health Organization (WHO) alcohol drinking guidelines, a woman should not drink more than two drinks per day, a man should not drink more than three drinks per day, and 1 day per week should be alcohol-free.

#### Physically Active

Participants engaged in moderate physical activity, including walking, for at least 150 min/week and fewer than 150 min/week were considered to be physically inactive (26).

### Data Quality Assurance and Processing

The questionnaire was pretested on 5% of the sample size of hypertension patients who had follow-up at Felege Hiwot comprehensive specialized hospital. The questionnaire was modified to enhance the consistency of understanding by the respondents as well as by the data collector. The data was collected by 3 trained physiotherapists. The data collectors were trained by the primary investigator about the general purpose of the study and data collection procedures. The questionnaire was translated from English to Amharic by language experts, then translated back to English during data analysis.

### Data Analysis

Data were cleaned, coded and entered Epi info version 7.2.1 and exported to SPSS version 23.0. Descriptive statistics were summarized in percentage and frequency. The statistical analysis using binary logistic regression was done to determine the candidate variables for multivariable logistic regression at *p*-value < 0.2. Then, variables having *p*-value < 0.05 at 95% in multivariable logistic regression were statistically significant.

### Ethical Consideration

Ethical clearance was obtained from the ethical review committee of the school of medicine under the delegation of the University of Gondar Institutional review board. Before data collection, the purpose of this study, potential indirect benefit, and right to refuse were explained to each participant. After that, written informed consent is taken from each study participant. Information was recorded anonymously, and confidentiality was assured throughout the data collection.

## Results

### Socio Demographic Characteristics of Hypertensive Patients

Out of the total 424 study participants, 393 participated in the study, with a response rate of 92.7%. The age of the participants ranged from 27 to 93 years, with a mean age of 57.6 (SD 11.39) years. More than half of the participants were female, and nearly half of the participants were found between the ages of 50 and 65. The majority of the participants (71.5%) resided in the urban areas (Table 1).

**Table 1:** Socio-demography of participants attending in chronic illness follow-up Clinic of University of Gondar Comprehensive Specialized Hospital, Northwest, Ethiopia, 2021 (*n* = 393).

**Variables**	**Category**	**Frequency (%)**
sex	Male	163 (41.5)
	Female	230 (58.5)
Age	<50	116 (29.5)
	50–65	179 (45.5)
	>65	98 (24.9)
Religion	Orthodox	347 (88.3)
	Muslim	46 (11.7)
Residence	Rural	112 (28.5)
	Urban	281 (71.5)
Marital status	Married	253 (64.4)
	Unmarried	140 (35.6)
Educational status	No formal education	192 (48.9)
	Primary education	90 (22.9)
	High school and above	111 (28.2)
Occupational status	Farmer	32 (8.1)
	Housewife	155 (39.4)
	Private employee	104 (26.5)
	Government employee	42 (10.7)
	Retired	60 (15.3)
Family income	≤5,000	359 (91.3)
	>5,000	34 (8.7)

### Clinical and Behavioral Characteristics of Hypertensive Patients

Almost half (49.4%) of the participants had a history of hypertension for the last 5 years, and more than one third of the participants had concomitant diabetes mellitus. Nearly one third of the participants knew someone with one stroke and was physically active (Table 2).

**Table 2:** Clinical and behavioral characteristics of participants attending in chronic illness follow-up Clinic of University of Gondar Comprehensive Specialized Hospital, Northwest, Ethiopia, 2021 (*n* = 393).

**Variables**	**Category**	**Frequency (%)**
Diabetes Mellitus	Yes	147 (37.4)
	No	246 (62.6)
Duration of hypertension	<5	199 (50.6)
	≥5	194 (49.4)
Heart disease	Yes	36 (9.2)
	No	357 (90.8)
Family history of stroke	Yes	31 (7.9)
	No	362 (92.1)
Knowing some with stroke	Yes	137 (34.9)
	No	256 (65.1)
Smoking	Yes	11 (2.8)
	No	382 (97.2)
Alcohol drinking	Yes	60 (15.3)
	No	333 (84.7)
Physically active	Yes	125 (31.8)
	No	268 (68.2)

### General Knowledge of Hypertensive Patients About Stroke

More than half of the participants 220 (56%) have never heard of stroke, and only a quarter of the participants 99 (25.2%) regraded stroke is a brain disease, while approximately 57 (14.5%) believed in homeopathic treatment for stroke. Some misconceptions have been observed, such as 21 (5%) believing that stroke was contagious, while 43 (10.9%) believed that it was caused by sin. Only 133 (33.8%) agreed that e stroke can be avoided. While one-third of 130 people (33.1%) accepted that stroke was a disease in the Elderly.

### Knowledge of the Warning Signs of Stroke Among Hypertensive Patients

More than half the participants (58.1%) had no any stroke warning signs. The warning signs of stroke identified by one third of the participants (37.4%) as sudden onset of half body weakness, followed by sudden onset of loss of consciousness (27.7%), sudden onset of speech problems (22.4%), sudden onset of memory loss (19.4%), and sudden onset of double vision (18.6%) (Table 3).

**Table 3:** Stroke warning sign/symptom of patients attending in chronic illness follow-up Clinic of University of Gondar Comprehensive Specialized Hospital, Northwest, Ethiopia, 2021 (*n* = 393).

**Variables**	**Categories**	**Frequency (%)**
sudden onset of dizziness	Yes	67 (17)
	No	326 (83)
Sudden onset of headache	Yes	56 (14.2)
	No	337 (85.8)
Sudden onset of memory loss	Yes	77 (19.4)
	No	316 (80.4)
Sudden onset of half body weakness	Yes	147 (37.4)
	No	246 (62.6)
Sudden onset of loss of consciousness	Yes	109 (27.7)
	No	284 (72.3)
Sudden onset of double vision	Yes	73 (18.6)
	No	320 (81.4)
Sudden onset of speech problems	Yes	88 (22.4)
	No	305 (77.6)

### Knowledge of Risk Factors of Stroke Among Hypertensive Patients

Nearly half of the participants (46.1%) didn't know any of the listed stroke risk factors. The common risk factors identified by the study participants were hypertension (48.1%), followed by alcohol consumption (38.2%), hypercholesterolemia (36.1%), physical inactivity (33.8%), obesity (33.3%), smoking (28%), and diabetes mellitus (23.2%) (Table 4).

**Table 4:** Stroke risk factors of patients attending in chronic illness follow-up Clinic of University of Gondar Comprehensive Specialized Hospital, Northwest, Ethiopia, 2021 (*n* = 393).

**Variables**	**Category**	**Frequency**
High blood pressure	Yes	189 (48.1)
	No	204 (51.8)
Smoking	Yes	110 (28)
	No	283 (72)
Diabetes mellitus	Yes	91 (23.2)
	No	302 (76.8)
Cardiac disease	Yes	81 (20.6)
	No	312 (79.4)
Obesity	Yes	131 (33.3)
	No	262 (66.7)
High Cholesterol	Yes	142 (36.1)
	No	251 (63.9)
Excessive alcohol intake	Yes	150 (38.2)
	No	243 (61.8)
Physically active	Yes	133 (33.8)
	No	260 (66.2)
Presence of family member having stroke	Yes	61 (15.5)
	No	332 (84.5)

### Knowledge of Hypertensive Patients Toward Stroke

Out of 393 participants, 40.7% (160) (95% CI: 35.9, 45.5) of the study participants had adequate knowledge toward stroke warning signs and risk factors.

### Prevention Practice of Hypertensive Patients Toward Stroke

In our study, 51.7% (95% CI: 46.8, 56.5) of the participants had good stroke prevention practice. The majority of the participants avoided or quite smoking and reduced alcohol consumption by 97.7 and 89.6%, respectively, whereas almost three-quarters of the participants reduced salt consumption (72%) and attended their regular clinic follow-up (75%). However, only 19.8% consumed fruits and vegetables, 36.6% engaged in regular physical activity, and 42.2% avoided fatty foods (Table 5).

**Table 5:** Stroke prevention practice of patients attending in chronic illness follow-up Clinic of University of Gondar Comprehensive Specialized Hospital, Northwest, Ethiopia, 2021 (*n* = 393).

**Variables**	**Category**	**Frequency (%)**
Attend follow up at clinic	Yes	297 (75.6)
	No	96 (24.4)
Taking the prescribed medications adherently	Yes	268 (68.2)
	No	125 (31.8)
Check blood pressure regularly	Yes	239 (60.8)
	No	154 (39.2)
Regular physical activity	Yes	145 (36.9)
	No	248 (63.1)
Regularly consume fruits and vegetables	Yes	78 (19.8)
	No	315 (80.2)
Avoid fatty foods	Yes	166 (42.2)
	No	227 (57.8)
Avoid or quit smoking	Yes	384 (97.7)
	No	9 (2.3)
Reduce alcohol intake	Yes	352 (89.6)
	No	41 (10.4)
Reduce salt consumption	Yes	283 (72)
	No	110 (28)

### Factors Associated With Knowledge of Hypertensive Patients Toward Stroke

Sex, age, educational status, occupation, place of residence, household monthly income, knowing someone with stroke, and physical activity were entered into multivariable analysis. Adequate stroke knowledge was significantly associated with formal education, knowing someone with stroke and physical activity.

Educational status was directly related to stroke-related knowledge in hypertensive patients. Hypertensive patients with high school or above had 4.6 times the odds of having adequate stroke knowledge compared to those with no formal education [AOR = 4.6 (95% CI: 2.08, 10.17)]. Hypertensive patients who knew someone with a stroke had 13.17 times the odds of having adequate stroke knowledge compared to those hypertensive patients who didn't know anyone had a stroke [AOR = 13.17 (95% CI: 7.3, 23.77)]. Hypertensive patients who are physically active had 4.05 times the odds of having adequate stroke knowledge compared to those who are physically inactive [AOR = 4.05 (95% CI: 2.23, 7.37)] (Table 6).

**Table 6:** Factors associated with stroke knowledge in hypertensive patients attending in chronic illness follow-up clinic of University of Gondar Comprehensive Specialized Hospital, Northwest, Ethiopia, 2021 (*n* = 393).

**Variables**	**Categories**	**Stroke knowledge**		**COR (95% CI)**	**AOR (95% CI)**
		**Good**	**Poor**		
sex	Male	73 (44.8.)	90 (55.2)	Ref	Ref
	Female	87 (37.8)	143 (62.2)	0.75 (0.5,1.13)	1.28 (0.59, 2.76)
Age	<50	37 (31.9)	79 (68.1)	**Ref**	
	50–65	84 (46.9)	95 (53.1)	1.88 (1.15,3.07)	1.22 (0.625,2.36)
	>65	39 (39.8)	59 (60.2)	1.41 (0.80, 2.47)	1.74 (0.0.76, 4.0)
Educational status	No formal education	49 (25.5)	143 (74.5)	**Ref**	Ref
	Primary education	31 (34.4)	59 (65.6)	1.53 (0.89, 2.63)	1.83 (0.89, 3.74)
	Secondary education and above	80 (72.1)	31 (27.9)	7.53 (4.45, 12.75)	4.6 (2.08, 10.17) ^**^
Occupation	Housewife	41 (71.9)	16 (28.1)	10.54 (4.16,26.69)	2.8 (0.85, 9.26)
	Farmer	28 (66.7)	14 (33.3)	8.22 (3.12, 21.7)	1.35 (0.34, 5.33)
	Government	32 (30.8)	72 (69.2)	1.83 (0.79,4.23)	0.65 (0.22, 1.85)
	Private	50 (34.7)	94 (65.3)	2.19 (0.98, 4.89)	1.41 (0.53, 3.75)
	Retired	9 (19.6)	37 (80.4)	Ref	ref
Place of residence	Urban	132 (47)	149 (53)	2.65 (1.63, 4.32)	1.046 (0.52, 2.1)
	Rural	28 (25)	84 (75)	**Ref**	Ref
Household monthly income	≤ 5,000	135 (37.6)	224 (62.4)	**Ref**	Ref
	>5,000	25 (73.5)	9 (26.5)	4.60 (2.08, 10.16)	1.05 (0.32, 3.48)
Knowing someone with stroke	Yes	110 (80.3)	27 ((19.7)	16.78 (9.95,28.29)	13.17 (7.3, 23.77) ^**^
	No	50 (19.5)	206 (80.5)	1	1
Physical activity	Yes	80 (64)	45 (36)	4.18 (2.66, 6.55)	4.05 (2.23, 7.36) ^**^
	No	80 (29.9)	188 (70.1)		

* *Significant at p-value ≤ 0.05*,

** *Significant at p-value ≤ 0.001*.

### Factors Associated With Prevention Practice of Hypertensive Patients Toward Stroke

Like knowledge, multiple variables found to be affected the stroke prevention practice of hypertensive patients. Educational status, place of residence, duration of hypertension, knowing someone with stroke and physical activity were significantly associated with good stroke prevention practice in multivariable logistic regression.

Hypertensive patients with primary education [AOR = 2.61 (95% CI: 1.44, 4.73)] and secondary or higher education [AOR = 3.75 (95% CI: 1.99, 7.05)] had 2.61 times and 3.75 times respectively, the odds of engaging in good stroke prevention practices compared to hypertensive patients with no formal education. Hypertensive patients residing in urban areas had 9.65 times the odds of engaging in good stroke prevention practices compared to those living in rural areas [AOR = 9.65 (95% CI: 5.04, 18.44)]. Hypertensive patients who had <5 years of duration of hypertension had 1.9 times the odds of engaging in good stroke prevention practices compared to participants who had 5 year or above duration of hypertension (AOR = 1.9, 95% CI:1.15,3.14).

Hypertensive patients who knew someone with a stroke had 2.27 times the odds of engaging in good stroke prevention practices compared to those hypertensive patients who didn't know anyone had a stroke (AOR = 2.27:95% CI: 1.3, 3.93). Hypertensive patients who are physically active had 1.76 times the odds of engaging in good stroke prevention practices compared to those who are physically inactive (AOR = 1.76, 95% CI: 1.03, 3.01) (Table 7).

**Table 7:** Factors associated with stroke prevention practices in hypertensive patients attending in chronic illness follow-up clinic of University of Gondar Comprehensive Specialized Hospital, Northwest, Ethiopia, 2021 (*n* = 393).

**Variables**	**Categories**	**Stroke prevention practice**		**COR (95% CI)**	**AOR (95% CI)**
		**Good**	**Poor**		
Age	<50	52 (44.8)	64 (55.2)	1	1
	50–65	100 (55.9)	79 (44.1)	1.56 (0.97, 2.5)	0.918 (0.49, 1.69)
	>65	51 (52)	47 (48)	1.34 (0.78, 2.29)	1.02 (0.48, 2.18)
Education	No formal education	62 (32.3)	130 (67.7)	1	1
	Primary education	51 (56.7)	39 (43.3)	2.74 (1.63, 4.6)	2.61 (1.44, 4.73)^*^
	Secondary education and above	90 (81.1)	21 (18.9)	8.99 (5.12,15.8)	3.75 (1.99, 7.05) ^*^
Occupation	Housewife	38 (66.7)	19 (33.3)	5.078 (2.18, 11.83)	0.50 (0.16, 1.55)
	Farmer	33 (78.6)	9 (21.4)	9.31 (3.503, 24.75)	0.56 (0.15,2.10)
	Government employee	55 (52.9)	49 (47.1)	2.85 (1.348, 6.022)	0.74 (0.28, 1.94)
	Private	64 (44.4)	80 (55.6)	2.03 (0.98, 4.18)	0.80 (0.32, 2.01)
	Retired	13 (28.3)	33 (71.7)	1	
Residence	Urban	188 (66.9)	93 (33.1)	13.07 (7.19, 23.77)^**^	9.65 (5.04, 18.44) ^**^
	Rural	15 (13.4)	97 (86.6)	1	1
Family income	≤ 5,000	181 (50.4)	178 (49.6)	1	1
	>5,000	29 (85.3)	5 (14.7)	4.01 (1.703, 9.45)	0.83 (0.29, 2.34)
Duration of HTN	<5 years	116 (58.3)	83 (41.7)	1.0.34 (0.903, 1.99)	1.9 (1.15, 3.14) ^*^
	≥5 years	94 (48.5)	100 (51.5)	1	1
Knowing someone with stroke	Yes	97 (70.8)	40 (29.2)	3.1 (1.99, 4.8)	2.27 (1.30, 3.93) ^*^
	No	113 (44.1)	143 (55.9)	1	1
Smoking habit	Yes	9 (81.8)	2 (18.2)	4.36 (0.93, 20.5)	1.20 (0.22, 6.43)
	No	201 (52.6)	181 (47.4)	1	1
Physically active	Yes	87 (69.6)	38 (30.4)	2.57 (1.64, 4.001)	1.76 (1.03, 3.01) ^*^
	No	123 (45.9)	145 (54.1)	1	1

* *Significant at p value ≤ 0.05*,

** *Significant at p value ≤ 0.001*.

## Discussion

The study aimed to assess the knowledge of stroke, prevention practice and associated factors among patients with hypertension in the University of Gondar comprehensive specialized hospital, Gondar, Ethiopia.

The finding of this study revealed that more than one third of the participants (40.7%) had adequate knowledge about stroke, which is higher than the finding of previous studies in Ethiopia [Debre-Tabor, 24.9 % (27), 15% (28), Bahir dar, 18.3% (26)], and India, 24% (30). The possible explanation for differences in the prevalence of stroke knowledge among hypertensive patients could be the criteria used to define stroke knowledge. In the study done in Bahir Dar, the number of warning signs and risk factors mentioned were used to define adequate stroke knowledge, whereas in the study done in Debre Tabor, the stroke knowledge was measured by the stroke warning signs (28) and prevention practices (27) separately. However, this finding is lower than the study conducted in Nigeria 70.3%) (14), India, 66.7% (31). This could be due to a difference in the socio-demography of the study populations. One of the possible reasons might be the fact that only half of the participants in our study had formal education, whereas three-quarters of the participants in a study done in Nigeria had formal education.

More than half (51.7%) of the participants had good prevention practice for stroke. In contrast with the previous literature, a higher prevalence of good stroke prevention practice than stroke knowledge has been reported in our study. A possible explanation could be that hypertensive patients may be exposed to health education regarding common non-communicable diseases, including stroke, which have similar prevention practices, but they may not identify the specific prevention methods for stroke.

The most recognized warning signs of stroke in our study were the sudden occurrence of half body weakness, loss of consciousness, and speech problems. Participants also identified hypertension as a risk factor for stroke. Alcohol consumption and hypercholesterolemia were the subsequent risk factors identified. This is in agreement with the study done in Morocco where the most frequently identified warning signs and risk factors were sudden weakness of the arm, leg or face and hypertension, respectively (25).

In multivariable logistic regression analysis; higher educational status, knowing someone with stroke and being physically active were significantly associated with good stroke knowledge.

In the present study, education was significantly associated with having adequate stroke knowledge. Hypertensive patients who had secondary education, and above were more than four times more likely to have adequate stroke knowledge. This was concordant with the findings of a range of studies in which high educational level had been the most associated factor with adequate knowledge of stroke in hypertensive patients in Ethiopia (26, 28, 29), and Nigeria (14, 32) Morocco (25), and Beirut (33). This may be due to the fact that individuals who have a higher educational level may have more interaction with society and have easy access to health-related information from various sources like the internet, news, books, magazines, and literature as compared with individuals who have no formal education, which is supported by the systematic reviews reporting that educational status has a significant impact on cardiovascular disease knowledge in SSA populations (24, 34). Furthermore, a similar study conducted on the general population revealed that a low educational level is associated with poor stroke knowledge (35).

In this study, knowing someone with a stroke was one of the predictors of stroke knowledge among hypertensive patients. Those who knew someone who had a stroke were 13.17 times more likely than their counterparts to have adequate stroke-related knowledge. This is consistent with the findings of studies conducted in Addis Abeba, Ethiopia (36), Nigeria (32) and Morocco (25). However, this finding is inconsistent with the findings of a study conducted on hypertensive patients in Pakistan, which found that knowing someone who had a stroke was not associated with knowing about stroke risk factors or warning signs (37). The odds of hypertensive patients who knew someone who had a stroke were 2.27 times more likely to engage in good stroke prevention practice than those of hypertensive patients who didn't know anyone had a stroke. A possible explanation could be that family, friends and the community could be the primary sources of information regarding strokes. This is supported by the study done in South Asian populations, which found that lack of reliable sources of information was identified as a significant determinant factor for adequate knowledge of stroke.

This study revealed that the odds of physically active participants having adequate knowledge of stroke were 4.06 times higher compared to those who were physically inactive. Physically active participants were 1.76 times more likely than physically inactive participants to have adequate stroke preventive practices. Our findings are supported by the report from Hungary (38). A possible explanation could be that health-conscious people are more likely to engage in beneficial health behaviors like regular exercise because they tend to have a better understanding of their health, pay attention to individual health problems, and then take healthy measures to ensure their personal health. Having a higher educational level, being an urban resident, knowing someone with a stroke, and being physically active were significantly associated with good stroke prevention practice.

Participants with higher educational status were more likely to have good stroke prevention practice. Participants who completed primary and high school and above had a higher stroke prevention practice than study participants who had no formal education by 2.61 and 3.75, respectively. A similar finding was reported by a systematic review which reported that poor stroke prevention practices were related to low educational levels (24). The explanation could be that patients who have at least completed primary education may have a better chance of exposure to different communication media like magazines, leaflets, books, and the internet. The other predictor of stroke prevention practices was residence. Urban residences were 9.65 times more likely to have good stroke prevention practice than those in rural areas. This could explain why the majority of educated hypertensive patients live in cities.

## Limitation of the Study

Since the study was institutional based and excluded hospitalized patients, generalization of the findings to the general population is limited; Also, since it was confined to a public hospital, hypertensive patients with higher economic status and education may have had follow-up at another private hospital and the study did not assess them. Furthermore, the study used close-ended questions. This might have limited the participant's responses regarding their knowledge and prevention practices. The hypertensive patient's attitudes toward stroke prevention were not assessed in this study.

## Conclusion

The proportion of participants with good stroke-related knowledge and prevention practice was good. Being physically active, having a higher educational level, and knowing someone who has had a stroke were all significantly associated with adequate stroke knowledge. While, having a formal education, knowing someone with a stroke, being an urban dweller, having a short duration of hypertension, and being physically active were significantly associated with good stroke prevention practices.

## Data Availability Statement

The raw data supporting the conclusions of this article will be made available by the authors, without undue reservation.

## Ethics Statement

The studies involving human participants were reviewed and approved by Ethical Review Committee of the School of Medicine under the delegation of the University of Gondar Institutional Review Board. The patients/participants provided their written informed consent to participate in this study.

## Author Contributions

FKW contributed to the conception of the research, study design, data collection, data entering, analysis, interpretation of data, drafting the work, and writing and review of the manuscript. GAE contributed to the conception, study design, analysis, and writing and review of the manuscript. TK, WB, AKK, and YGG contributed to the study design, analysis, and review of the research. All the authors read and approved the final manuscript to be submitted for publication.

## Funding

This research has been funded by the University of Gondar, Gondar, Ethiopia.

## Conflict of Interest

The authors declare that the research was conducted in the absence of any commercial or financial relationships that could be construed as a potential conflict of interest.

## Publisher's Note

All claims expressed in this article are solely those of the authors and do not necessarily represent those of their affiliated organizations, or those of the publisher, the editors and the reviewers. Any product that may be evaluated in this article, or claim that may be made by its manufacturer, is not guaranteed or endorsed by the publisher.
